# The impact of maternal SARS-COV-2 infection in early stages of newborn neurodevelopment: preliminary results in a multicenter Spanish study

**DOI:** 10.1192/j.eurpsy.2022.294

**Published:** 2022-09-01

**Authors:** Á. Castro Quintas, N. San Martín, I. De Las Cuevas, E. Eixarch, M. Daura-Corral, M. Lopez, L. De La Fuente Tomas, M.P. Garcia-Portilla, L. Fañanas, R. Ayesa-Arriola

**Affiliations:** 1 Centre for Biomedical Research Network on Mental Health (CIBERSAM), Instituto De Salut Carlos Iii, Madrid, Spain; 2 University of Barcelona, Evolutionary Biology, Ecology And Environmental Sciences, Barcelona, Spain; 3 University of Barcelona, Department Of Evolutive Biology, Ecology And Ambiental Sciences, Barcelona, Spain; 4 Universitary Hospital Marques de Valdecilla, Neonatology, Santander, Spain; 5 Maternitat Hospital Clinic Barcelona, Bcnatal Fetal Medicine Research Center, Barcelona, Spain; 6 University of Oviedo, Department Of Psychiatry, Oviedo, Spain; 7 Valdecilla Biomedical Research Institute, Psychiatry, Santander, Spain

**Keywords:** Newborn Development, Pregnancy Infection, COVID19, NBAS

## Abstract

**Introduction:**

The consequences for the COVID-19 pandemic in the newborns of affected mothers remains unknown. Previous clinical experiences with other infections during pregnancy lead to considered pregnant women and their offspring especially vulnerable for SARS-COV-2. That is, the underlying physiopathological changes caused by the infection (e.g. storm of cytokines, micro-coagulation in placenta or vertical transmission) could clearly compromise fetal neurodevelopment.

**Objectives:**

To analyze the impact of maternal SARS-COV-2 infection during pregnancy in early neurodevelopment of infants gestated during the COVID-19 pandemic period compared to those gestated immediately prior (2017-2021).

**Methods:**

212 pregnant women (14% infected) were followed throughout their pregnancy and postpartum, including newborn development. SARS-COV-2 infection was serologically confirmed during pregnancy. The Brazelton Neonatal Assessment Scale (NBAS) was administered at 6 weeks old by a trained neonatologist to evaluate neurological, social and behavioral aspects of newborn’s functioning. Differences in NBAS scores between cases and controls were tested by ANOVAs. All the analysis were adjusted for maternal age, sociodemographic status, anxious-depressive symptomatology, infant’s sex and gestational age at birth and NBAS, and for the period of gestation (previous or during COVID-19 pandemic).

**Results:**

NBAS social interactive dimension was significantly decreased in those infants exposed to prenatal SARS-COV-2 (F=4.248, p=.043), particularly when the infection occurred before the week 20 of gestation. Gestation during COVID-19 pandemic did not alter NBAS subscales.

**Conclusions:**

SARS-COV-2 infection during pregnancy seems to be associated with lower NBAS scores on social dimension in 6 weeks old exposed newborns.

**Disclosure:**

No significant relationships.

